# The Effects of Immersive Virtual Reality Applications on Enhancing the Learning Outcomes of Undergraduate Health Care Students: Systematic Review With Meta-synthesis

**DOI:** 10.2196/39989

**Published:** 2023-03-06

**Authors:** Justina Yat Wa Liu, Yue-Heng Yin, Patrick Pui Kin Kor, Daphne Sze Ki Cheung, Ivy Yan Zhao, Shanshan Wang, Jing Jing Su, Martin Christensen, Stefanos Tyrovolas, Angela Y M Leung

**Affiliations:** 1 School of Nursing The Hong Kong Polytechnic University Hong Kong Hong Kong; 2 Research Institute for Smart Ageing The Hong Kong Polytechnic University Hong Kong Hong Kong; 3 School of Nursing Nanjing Medical University Nanjing China

**Keywords:** immersive virtual reality, virtual reality, effects, undergraduate health care education, systematic review, meta-synthesis

## Abstract

**Background:**

Immersive virtual reality (IVR) applications are gaining popularity in health care education. They provide an uninterrupted, scaled environment capable of simulating the full magnitude of sensory stimuli present in busy health care settings and increase students’ competence and confidence by providing them with accessible and repeatable learning opportunities in a fail-safe environment.

**Objective:**

This systematic review aimed to evaluate the effects of IVR teaching on the learning outcomes and experiences of undergraduate health care students compared with other teaching methods.

**Methods:**

MEDLINE, Embase, PubMed, and Scopus were searched (last search on May 2022) for randomized controlled trials (RCTs) or quasi-experimental studies published in English between January 2000 and March 2022. The inclusion criteria were studies involving undergraduate students majoring in health care, IVR teaching, and evaluations of students’ learning outcomes and experiences. The methodological validity of the studies was examined using the Joanna Briggs Institute standard critical appraisal instruments for RCTs or quasi-experimental studies. The findings were synthesized without a meta-analysis using vote counting as the synthesis metric. A binomial test with *P*<.05 was used to test for statistical significance using SPSS (version 28; IBM Corp). The overall quality of evidence was evaluated using the Grading of Recommendations Assessment, Development, and Evaluation tool.

**Results:**

A total of 17 articles from 16 studies totaling 1787 participants conducted between 2007 and 2021 were included. The undergraduate students in the studies majored in medicine, nursing, rehabilitation, pharmacy, biomedicine, radiography, audiology, or stomatology. The IVR teaching domains included procedural training (13/16, 81%), anatomical knowledge (2/16, 12%), and orientation to the operating room setting (1/16, 6%). The quality of the 75% (12/16) of RCT studies was poor, with unclear descriptions of randomization, allocation concealment, and outcome assessor blinding procedures. The overall risk of bias was relatively low in the 25% (4/16) of quasi-experimental studies. A vote count showed that 60% (9/15; 95% CI 16.3%-67.7%; *P*=.61) of the studies identified similar learning outcomes between IVR teaching and other teaching approaches regardless of teaching domains. The vote count showed that 62% (8/13) of the studies favored using IVR as a teaching medium. The results of the binomial test (95% CI 34.9%-90%; *P*=.59) did not show a statistically significant difference. Low-level evidence was identified based on the Grading of Recommendations Assessment, Development, and Evaluation tool.

**Conclusions:**

This review found that undergraduate students had positive learning outcomes and experiences after engaging with IVR teaching, although the effects may be similar to those of other forms of virtual reality or conventional teaching methods. Given the identification of risk of bias and low level of the overall evidence, more studies with a larger sample size and robust study design are required to evaluate the effects of IVR teaching.

**Trial Registration:**

International prospective register of systematic reviews (PROSPERO) CRD42022313706; https://www.crd.york.ac.uk/prospero/display_record.php?RecordID=313706

## Introduction

### Background

Clinical competency is essential for all health care professionals. Health care students, particularly undergraduate students with limited clinical experience, must incorporate theories into their skills with a good professional attitude to attain clinical competency. In addition to conventional classroom teaching for theoretical knowledge inputs and laboratory skill practice, the clinical practicum is crucial in undergraduate health care education to develop the clinical competency of students [1]. However, clinically based degree programs such as medicine, nursing, and physiotherapy are facing faculty shortages and increasing demands on clinical venues for student clinical placements [2]. In addition, clinical practicums for undergraduate health care students have been suspended in many countries because of the COVID-19 pandemic, further diminishing learning opportunities for students [3]. Therefore, there is an ongoing need for accessible, cost-effective, and high-quality methods of education to overcome these resource limitations [2].

Simulation-based learning is useful for teaching in all domains (ie, knowledge, skills, and attitudes) relevant to the education of health professionals [4]. It is a well-applied teaching and learning strategy [5] for increasing training opportunities and enhancing learning efficiency [6]. Simulation-based learning facilitates a deeper understanding of theoretical knowledge, relationships between different concepts, advanced inquiry, problem-solving, and decision-making [7]. It allows health care students to practice procedural skills without compromising patient safety and improves the quality of their patient care [8]. Many studies have shown that simulation-based learning effectively advances students’ competencies in acquiring diagnostic and psychomotor skills [9,10]. However, it is not aimed at replacing the clinical practicum but at better engaging and preparing students for it.

Immersive virtual reality (IVR) applications for simulation-based training are gaining popularity in health care professional education [2,11]. They provide an immersive learning experience with a first-person viewpoint in a 3D virtual environment supported by head-mounted displays or Cave Automatic Virtual Environments (room-sized cube virtual reality [VR] environments) [12,13]. They provide direct sensory feedback or reactions to users based on their physical actions.

Compared with nonimmersive VR (such as computer-based simulation games) or other forms of traditional simulation-based training (such as skill laboratories using mannequins and simulators), only IVR provides learners with the perception of being physically present in a synthetic world [14]. It provides an uninterrupted, scaled environment capable of simulating the full magnitude of sensory stimuli present in busy health care settings such as operating rooms, emergency departments, and surgical or medical wards [11]. Owing to the uniquely high level of immersion that IVR offers, students can practice a particular procedure or rehearse a specific action with a high level of physical and psychological fidelity, with immediate and standardized feedback provided corresponding to the users’ actions. IVR can increase the competence and confidence of students by providing them with accessible and repeatable learning opportunities in a fail-safe environment [2].

The COVID-19 pandemic has led to a paradigm shift in all clinically based undergraduate programs, prompting adaptations through better use of educational technology, in which IVR is a promising alternative [15]. It is believed that the use of IVR-based simulation learning in health care professional training will persist beyond the pandemic [16]. A small number of systematic reviews have reported the efficacy of VR-based simulation learning in improving the knowledge and skill competence of students compared with conventional simulation-based training [1,17,18]. However, the learning approaches included in these reviews were mainly non-IVR approaches. Recently, a systematic review of 17 studies involving 307 surgical trainees reported significantly improved procedural completion times and greater postintervention scores on procedural checklists compared with conventional teaching methods such as watching standardized surgical training videos [11]. As previously mentioned, simulation-based learning, including the use of IVR, should be useful for learning in different domains (knowledge, skills, and attitudes) [4]. However, the extent to which IVR applications are used for teaching in different learning domains and their effectiveness in undergraduate health care professional education remain unknown [2].

### Objectives

Therefore, the aim of this review was to evaluate the effects of IVR applications in improving the learning outcomes and experiences of undergraduate health care students compared with other teaching methods. Two review questions were devised: (1) What are the effects of IVR applications on improving students’ learning outcomes in different teaching domains (such as knowledge, skills, and attitudes) compared with other teaching methods? (2) What are the effects of IVR applications on enhancing the learning experiences of students (such as their level of satisfaction, perception of IVR innovation, and self-perceived competence and confidence) compared with other teaching methods?

## Methods

This systematic review was conducted following the PRISMA (Preferred Reporting Items for Systematic Reviews and Meta-Analyses; Multimedia Appendix 1) [19] and SWiM (Synthesis Without Meta-analysis) [20] guidelines. The review protocol was registered in PROSPERO (CRD42022313706).

### Eligibility Criteria

We used the Population, Intervention, Comparison, Outcome, and Study Design (PICOS) structure to define the eligibility criteria (Textbox 1).

**Inclusion and exclusion criteria.**

- Inclusion criteria
  Population: the participants were undergraduate students majoring in professional health care fields such as medicine, nursing, and physiotherapy who were included without regard to sex or race.
  Intervention: the teaching methods used in the studies focused on immersive virtual reality (IVR) applications using either fully immersive 360° IVR, such as head-mounted display products, or Cave Automatic Virtual Environment systems, such as HTC VIVE, Samsung Oculus Rift, or other similar programs.
  Comparison: we aimed to determine whether other teaching methods would have similar effects to IVR applications in professional health care education. For the purpose of comparison, we classified the different teaching methods into either active or passive comparison groups. The active comparison groups included other forms of simulation-based training such as nonimmersive virtual reality (VR), computer screen–based simulators, and real patient simulation. The passive control groups included mainly conventional teaching methods such as face-to-face lectures, tutorial classes, role-play, and reading materials.
  Outcome: we determined the effects of IVR applications on the learning outcomes (ie, the primary outcome) and experiences (ie, the secondary outcome) of students. The primary outcome refers to the change in the students’ theoretical knowledge and procedural skills, as reflected by any form of written examination or clinical skills such as communication skills, completion time, and error rate in a specific clinical procedure. The secondary outcome was the students’ learning experience, as assessed by their satisfaction levels, perception of IVR innovations, and self-perceived competence and confidence after receiving IVR.
  Study design: this review included randomized controlled trials and quasi-experimental studies published in English between January 2000 and March 2022 as IVR technology has developed enough to be used in health education since 2000 [17].
- Exclusion criteria
  Studies were excluded if their target population was postgraduate health care students or students of other subjects unrelated to health care. Studies that involved only other forms of VR, such as nonimmersive VR, or other types of simulation-based technologies, such as augmented reality and manikins, were excluded. Pilot and feasibility studies were also excluded.

### Information Sources

A combination of Medical Subject Headings and free-text terms was used to search through 4 databases, namely, MEDLINE, Embase, PubMed, and Scopus, for potentially relevant abstracts. In addition, hand searches were conducted by reviewing the reference lists of all papers selected for inclusion in this review from the electronic databases, Google Scholar, and hard copies in university libraries to identify any articles missed by the database search. An alert for updated articles in each database was set to avoid missing potential up-to-date studies.

### Search Strategy

Search strategies were developed according to the 2 primary concepts of this review: the use of IVR applications in health professional undergraduate education and their effectiveness in enhancing the learning outcomes and experiences of students. To identify studies that used IVR applications, we used search terms such as “Immersive Virtual Reality” or “Simulated environment” and “Simulation” and “Healthcare” and “Students” or “Undergraduates” or “Trainees” (Multimedia Appendix 2). These terms were revised appropriately for different databases.

### Selection Process

The search results were imported into the EndNote bibliographic software (version 20; Clarivate Analytics), and duplicate studies were removed. The titles and abstracts of all the identified studies were screened independently by 2 researchers (JYWL and YHY) to identify potentially relevant papers based on the review criteria. Both researchers compared the preliminary results of the review to reach an agreement. Full-text articles were then obtained and screened independently. Any disagreements between the reviewers were then discussed among the members of the research team to reach a consensus.

### Data Collection Process and Data Items

A specific data extraction matrix was created to collect information from each included study, including the author, year, title, country of origin, demographic data of the participants (age, sex, and type of health profession), methodological data (aims of the study, sample size, study design, educational innovation, and comparison groups), and outcome data (primary outcome, eg, examination scores; secondary outcome, eg, self-perceived competence and confidence). If any original study had been published in more than one paper, the information was extracted as 1 study based on the study protocol number. The authors of the primary studies were contacted when clarifications were required or if any information was missing. Data extraction was conducted independently by the same 2 researchers. Once complete, the results were compared, and discrepancies were resolved through discussion. A final extraction table was developed.

### Study Risk-of-Bias Assessment

The studies selected for this review were independently assessed for methodological validity by the reviewers involved in their selection using the Joanna Briggs Institute (JBI; University of Adelaide, Australia) standard critical appraisal instruments for randomized controlled trials (RCTs) or quasi-experimental studies.

We assessed the included RCTs based on the following 12 appraisal items: methods of randomization; treatment allocation and concealment; similarity of characteristics between groups at baseline; blinding procedures for participants, interventionists, and outcome assessors; whether the comparison groups were treated identically other than in the intervention of interest (ie, IVR applications); completeness of the follow-up (ie, if there was any bias because of missing data); consistency and reliability of the outcome measurements; and appropriateness of the statistical analysis and trial design (JBI). For example, to determine if there were any biases because of missing data, we checked whether there were differences between groups with regard to the loss to follow-up (numbers or proportions, reasons, any analysis of patterns of loss to follow-up, and their impact on the internal validity of the study). We assessed the included quasi-experimental studies based on the following 9 appraisal items: clarity of dependent and independent variables, similarity of characteristics between groups at baseline, whether comparison groups were treated identically other than in the intervention of interest (ie, IVR applications), any comparison group, any pre- and postoutcome measurements, completeness of the follow-up (ie, whether there was any bias because of missing data), consistency and reliability of the outcome measurements, and appropriateness of the statistical analysis.

Working independently to assess for risk of bias, the same 2 assessors rated each item as *yes*, *no*, *unclear*, or *not applicable*. Any disagreements on the results of the bias assessment were then reviewed and discussed by the research team until a consensus was reached.

The aim of this assessment of the risk of bias was to determine the quality of each study. However, the risk of bias was not used as a criterion for the inclusion of a study in this review. A trial was judged to be at a low risk of bias overall when all items were rated as *yes*. Conversely, a study was judged to be at a high risk of bias when it reported on a procedure that could be judged as being a *no* or *unclear* in any item. Owing to the nature of the intervention, it was impossible to blind the participants; thus, we did not include the *blinding procedures for participants* item when determining a study’s overall risk of bias [21].

### Synthesis Methods

#### Criteria for Grouping Studies

Following the review questions, studies for synthesis were grouped according to the study outcomes (ie, learning outcomes for research question 1 and learning experience for research question 2). In addition, studies with the same teaching domains (ie, procedural skills vs theoretical knowledge) were grouped for the analysis of the 2 outcomes.

#### Standardized and Synthesis Metrics and Method

The direction of the effects (learning outcomes and learning experiences) was used as the standardized metric as there was a lack of precision, which was specific to the effects of the intervention (IVR teaching) and control on the results presented by different studies. This did not allow for the calculation of summary statistics [22]. In addition, the clinical and methodological characteristics (such as populations, intervention components, and the choice of outcome measurements and study designs) of each study were used to evaluate the heterogeneity based on the Cochrane Handbook for Systematic Reviews of Interventions [23]. The included studies were highly heterogeneous, with diverse teaching aims and outcome assessments to fit the needs of students from different health care professions. Different IVR features were adopted, with different frequencies and durations. In addition, most of the included studies were rated as having a high risk of bias. As a result, we felt that it was not appropriate to conduct a meta-analysis. Therefore, vote counting was the best match for synthesizing the results. A binomial test using SPSS (version 28; IBM Corp) was used to indicate whether there was evidence of an effect [23]. The quality of the evidence generated by different studies was assessed using the Grading of Recommendations Assessment, Development, and Evaluation (GRADE) tool [24].

## Results

### Search Results

**Figure 1:**
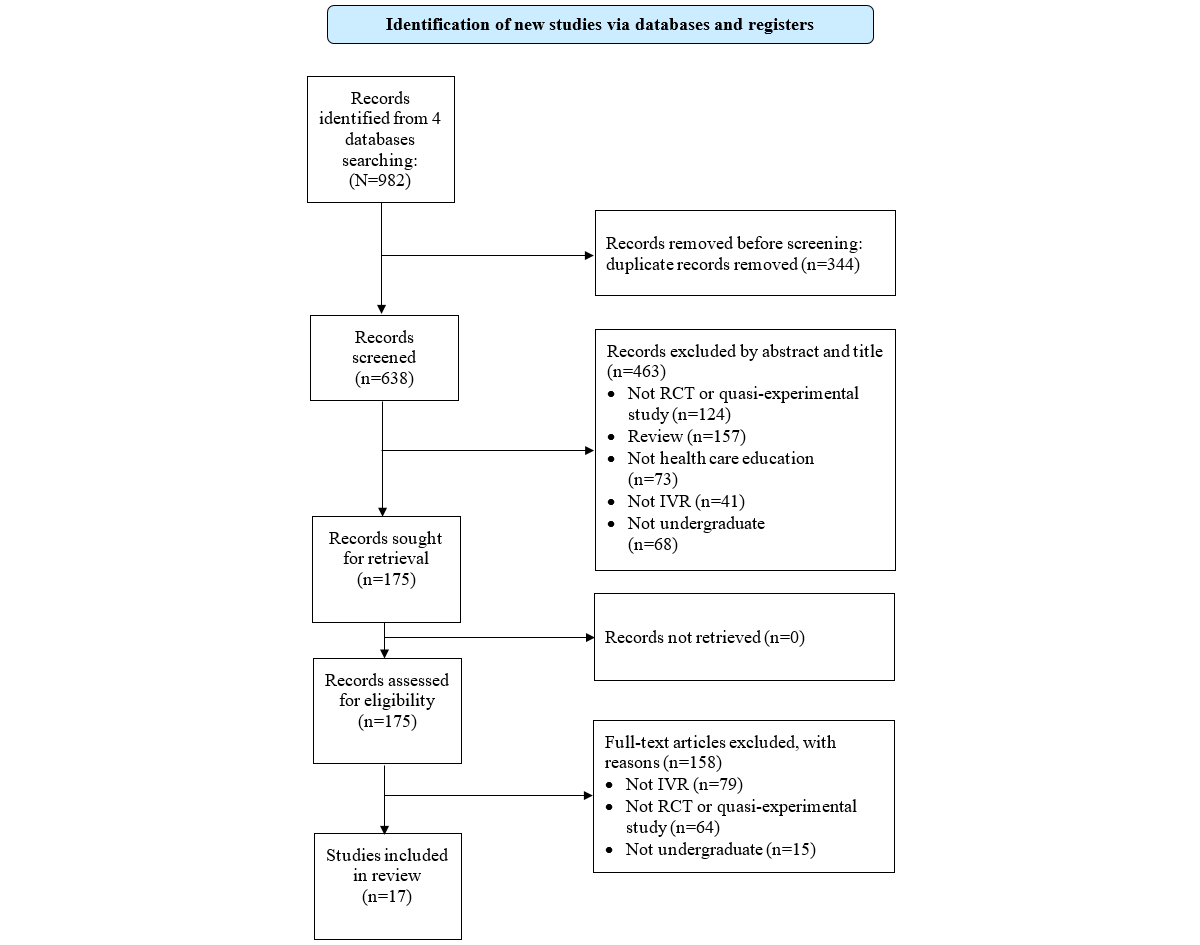
PRISMA (Preferred Reporting Items for Systematic Reviews and Meta-Analyses) flowchart of the literature screening process. IVR: immersive virtual reality; RCT: randomized controlled trial.

A total of 982 articles were found by searching the databases; after the removal of duplicates (n=344, 35%), 638 (65%) were left. After screening the titles and abstracts, 72.6% (463/638) of the articles were excluded, leaving 175 to be retrieved for a full-text screening. Following the full-text screening, a further 90.9% (159/175) of the articles were excluded, leaving 17 articles [25-41]. Of the 17 articles, 2 (12%) [31,32] were from the same study. Therefore, a total of 16 studies were included in this systematic review. Details of the selection process and the reasons for the exclusion of articles are presented in the PRISMA flowchart (Figure 1).

### Study Characteristics

The characteristics of the included studies are shown in Table 1. The 16 studies were conducted between 2007 and 2021. An RCT design was adopted in 75% (12/16) of the studies [25,26,28-30,32-34,36-38,40], and a quasi-experimental design was adopted in 25% (4/16) of the studies [27,35,39,41]. All 12 RCTs were 2-armed, among which were 8% (1/12) [26] that had a crossover design. Of the 4 quasi-experimental trials, 1 (25%) [39] was a 4-armed study, 1 (25%) [27] was a 3-armed study, and the other 2 (50%) were 2-armed studies [35,41].

A total of 1787 participants were involved in the included studies, with the number ranging from 25 to 289 in the RCTs and from 29 to 197 in the quasi-experimental studies. In total, 44% (7/16) of the studies involved sample sizes of <60. Female students accounted for 71.73%, with 25% (4/16) of the studies [25,32,35,39] not disclosing the sex distribution. The participants ranged in age from 19 to 35 years, whereas the age distribution was not discussed in 25% (4/16) of the studies [25,29,32,39]. The types of participants included undergraduate students majoring in medicine, nursing, rehabilitation, pharmacy, biomedicine, radiography, audiology, or stomatology.

IVR teaching was compared with other forms of VR such as desktop-based VR (ie, the active control) in 25% (4/16) of the studies [25,26,28,32]. In 50% (8/16) of the studies [29,30,34-38,40], IVR teaching was compared with conventional teaching methods such as verbal didactic instruction and hard-copy teaching materials (ie, the passive control). In total, 12% (2/16) of the studies [27,39] used passive and active controls. A total of 6% (1/16) of the studies [33] adopted role-play, and another study (1/16, 6%) [41] used a clinical practicum as their comparison group. These teaching approaches did not involve any use of VR, and we considered the role-play and clinical practicum groups as passive controls.

**Table 1:** Characteristics of the included studies (N=16).

Author, year, and country	Design	Sample size (percentage of female participants), N	Age (years)	Participants	Intervention (IVR^a^)	Comparator	Side effects
Gutiérrez-Maldonado et al [25], 2015, Spain	2-armed RCT^b^	Total: 52 (N/A^c^); EG^d^: 26; CG^e^: 26	N/A	Undergraduate students (without a specific discipline)	Training in simulated diagnostic interview techniques for eating disorders with an IVR application Number of IVR sessions: 1 Duration: allow students to complete the task Other teaching: a basic explanation by a professor before IVR Teaching domain: procedural skills	Training in simulated diagnostic interview techniques for eating disorders with a desktop-based VR^f^ Active control	N/A
Harrington et al [26,42], 2018, Ireland	2-armed crossover RCT	Total: 40 (50); EG: 20; CG: 20	23	Preclinical undergraduate students (medicine, physical therapy, pharmacy, and biomedical sciences)	Watching a 10-minute laparoscopic cholecystectomy operation with a 360° video using the IVR Number of IVR sessions: 1 Duration: 10 minutes Other teaching: none Teaching domain: procedural skills	Watching a 10-minute laparoscopic cholecystectomy operation video in 2D format on a 75-inch LED^g^ television Active control	Self-reported levels of nausea were low (0.7 out of 10) but varied substantially from 0 to 8 among participants.
Smith et al [27], 2018, United States	Quasi-experimental trial	Total: 197 (88.2); EG: 59; active CG: 58; passive CG: 55	18-25 (73.3%); 26-34 (15.5%); 35-50 (11.2%)	Senior baccalaureate-degree nursing students	Training in the skill of decontamination with IVR simulation using an HMD^h^ Number of IVR sessions: 1 Duration: allow students to complete the task Other teaching: 30-minute web-based video module to explain the skills Teaching domain: procedural skills	Training in the same skills with a case scenario in an emergency department room and featuring a patient on a stretcher, with available personal protective equipment and tools Active CG: PC and keyboard or mouse Passive CG: written instructions	None
Zackoff et al [28], 2020, United States	2-armed RCT	Total: 168 (48.8); EG: 78; CG: 90	20-24 (34.5%); 25-29 (60.1%); 30-34 (4.2%); 35-39 (0.6%)	Year-3 medical students	In addition to receiving the same 60-minute training session as in the CG, participants received a 30-minute IVR curriculum with 3 simulations of an infant from no distress to respiratory failure Number of IVR sessions: 1 Duration: 30 minutes Other teaching: facilitation and debriefing during and after the IVR tutorial Teaching domain: procedural skills	Receiving a 60-minute session consisting of verbal didactic instructions with a subsequent high-fidelity mannequin simulation to teach participants how to identify infants with respiratory distress and failure Active control	None
Francis et al [29], 2020, United States	2-armed RCT	Total: 52 (89); EG: 26; CG: 26	≤23 (40.4%); ≥24 (59.6%)	Year-2 preclinical physician assistant students	Attending a 5-minute IVR experience in the simulated operating room with several distinct scenarios. The IVR operating room scenario was viewed with a stand-alone untethered all-in-one VR headset. Number of IVR sessions: 1 Duration: 5 minutes Other teaching: none Teaching domain: orientation	Attending a traditional lecture on orientation to surgical operating room settings Passive control	N/A
Kurul et al [30], 2020, Turkey	2-armed RCT	Total: 72 (72.2); EG: 36; CG: 36	19	Year-1 physical therapy students	Learning head and neck region anatomy for 30 minutes with IVR Number of IVR sessions: 1 Duration: 30 minutes Other teaching: none Teaching domain: theoretical knowledge	Attending a 30-minute presentation of images of the head and neck region, use of computer-based VR Passive control	Vision discomfort (n=7), eyestrain (n=6), and general discomfort (n=6)
Gutiérrez et al [32], 2007, and Pierce et al [31], 2008, United States	2-armed RCT	Total: 25 (N/A); EG: 13; CG: 12	N/A	Year-1 medical students	Practicing how to conduct a physical examination on a patient with traumatic head injury in an IVR environment, which was displayed with an HMD Number of IVR sessions: 1 Duration: 30 minutes Other teaching: instructional video on using VR equipment and head injury reference materials Teaching domain: procedural skills	Practicing how to conduct a physical examination on a patient with traumatic head injury by using a computer screen and mouse to rotate the viewpoint, then using the joystick to perform a physical examination of the head injury Active control	None
Sapkaroski et al [33], 2019, Australia	2-armed RCT	Total: 76 (75); EG: 38; CG: 38	21	Radiography students	Practicing how to position a virtual avatar patient for PA^i^ imaging of the left hand by using CESTOL^j^ VR Clinic Number of IVR sessions: 1 Duration: allow students to complete the task Other teaching: hand imaging lesson Teaching domain: procedural skills	Practicing how to position the patient for PA imaging of the left hand using conventional clinical role-play in the x-ray laboratory Passive control	None
Stepan et al [34], 2017, United States	2-armed RCT	Total: 66 (50); EG: 33; CG: 33	21-25 (89.4%); 26-30 (9.1%); 31-35 (1.5%)	Year-1 and year-2 medical students	Learning neuroanatomy with a 10-minute IVR model using an HMD Number of IVR sessions: 1 Duration: 10 minutes Other teaching: 10-minute internet-based introductory lecture Teaching domain: theoretical knowledge	Independently studying neuroanatomy using web-based textbooks containing texts and 2D images for 20 minutes Passive control	None
Bakhos et al [35], 2020, France	Crossover quasi-experimental trial	Total: 29 (N/A); EG: 15; CG: 14	EG: 22; CG: 20	Year-1 audiology students (without beginning an internship)	Receiving a 3-hour IVR audiometry training session on 3 clinical cases (ie, presbycusis, vestibular schwannoma, and sudden idiopathic deafness). The audiometric diagnosis and management were evaluated for each case, and a report was generated that summarized the errors during the evaluation. Number of IVR sessions: 3 cases Duration: 30 minutes per case Other teaching: 20 minutes introducing the system and debriefing Teaching domain: procedural skills	Receiving a 3-hour audiometry training session supervised by a teacher on basic audiometry principles and practicing audiometry techniques for different clinical cases Passive control	None
Chao et al [36], 2021, Taiwan	2-armed RCT	Total: 45 (86.7); EG: 22; CG: 23	24	Nursing students (aged ≥20 years) who had never acquired the skills of NG^k^ tube feeding	Learning NG tube feeding skills through a 20-minute IVR video program Number of IVR sessions: 1 Duration: 20 minutes Other teaching: none Teaching domain: procedural skills	Learning the same skills by watching the NG tube feeding demonstration DVD video Passive control	A total of 5 students (23%) reported feeling slightly dizzy, but this did not affect their ability to watch the video.
Berg and Steinsbekk [37], 2020, Norway	2-armed RCT	Total: 289 (78.5); EG: 149; CG: 140	<20 (23.8%); 20-24 (64.6%); >25 (11.6%)	Year-1 medical and nursing students (who had started their studies no later than 2 months before this study)	Self-practicing the ABCDE^l^ approach in an IVR environment for assessing and managing patients who were critically ill or injured. All practice attempts were carried out on a virtual patient using virtual equipment in the IVR environment. Number of IVR sessions: for self-practicing, did not mention how many times the students could practice Duration: allow students to complete the task Other teaching: same as the comparator group except that they used traditional equipment to practice the skills Teaching domain: procedural skills	Self-practicing the ABCDE approach with traditional equipment after receiving a 1-hour teaching session, which included a 15-minute introduction, 20 minutes of individual practice, and 15 minutes of testing. All participants watched a 7-minute introduction video about the ABCDE approach. They received a printed sheet with pictures of the equipment along with simple instructions on its technical use. Passive control	N/A
Berg and Steinsbekk [38], 2021, Norway	2-armed RCT	Total: 289 (84.6); EG: 146; CG: 143	<20 (29.6%); 20-24 (59.3%); >25 (11.1%)	Year-1 medical and nursing students (who had started their studies no later than 2 months before this study)	Group practicing in the IVR platform in a virtual patient room using the ABCDE approach to immediately assess and treat patients who were critically ill or injured Number of IVR sessions: 1 Duration: allow students to complete the task Other teaching: 6-minute lecture and 7-minute skill-demonstration video Teaching domain: procedural skills	Group practicing with physical equipment using the ABCDE approach, receiving a printed sheet with pictures of the equipment along with simple instructions on its technical use Passive control	N/A
Collaço et al [39], 2021, Brazil	Quasi-experimental trial	Total: 163 (N/A); full: 42; NP^m^: 40; NT^n^: 40; NH^o^: 41	N/A	Clinical dental students	Receiving dental anesthesia skill training through an IVR application using an HMD. The training was divided into 2 phases: preceptorship and training. Number of IVR sessions: 2 Duration: did not mention Other teaching: none Teaching domain: procedural skills	NP: received nonimmersive VR in the preceptorship phase but IVR in the training phase. NT: received IVR in the preceptorship phase but nonimmersive VR in the training phase. NH: received nonimmersive VR in both phases. When under nonimmersive conditions, the participants visualized the preceptorship or performed the training by watching a television screen. Both passive and active controls	Most reported experiencing no side effects (87%) and “slight” (9%), “moderate” (3%), and “severe” (1%) symptoms.
Ros et al [40], 2020, France	2-armed RCT	Total: 173 (52); EG: 85; CG: 88	N/A	Year-4 medical students (2 years before residency)	Learning a medical procedure (ie, external ventricular drainage) through a 7-minute IVR displayed on an HMD Number of IVR sessions: 1 Duration: 7 minutes Other teaching: reading the technical note about this procedure Teaching domain: procedural skills	Reading only the technical note for 7 minutes, which described the procedure for external ventricular drainage Passive control	N/A
Yu et al [41], 2021, Korea	Quasi-experimental trial	Total: 51 (92); EG: 26; CG: 25	22.4	Senior nursing students	Learning high-risk neonatal infection control skills with 3 scenarios using an IVR simulation program plus clinical routine practice as the CG Number of IVR sessions: 1 Duration: allow students to complete the task Other teaching: prebriefing and debriefing before and after the IVR simulation Teaching domain: procedural skills	Having routine clinical practice in a neonatal intensive care unit Passive control	N/A

^a^IVR: immersive virtual reality.

^b^RCT: randomized controlled trial.

^c^N/A: not applicable.

^d^EG: experimental group.

^e^CG: control group.

^f^VR: virtual reality.

^g^LED: light-emitting diode.

^h^HMD: head-mounted display.

^i^PA: posterior-anterior.

^j^CESTOL: Clinical Education Training Solution.

^k^NG: nasogastric.

^l^ABCDE: airways, breathing, circulation, disability, exposure.

^m^NP: nonpreceptorship.

^n^NT: nontraining.

^o^NH: nonhaptic feedback.

### IVR Teaching Characteristics

A total of 81% (13/16) of the studies used IVR to train students in skills, including in techniques for diagnosing eating disorders [25] and respiratory distress in infants [28], laparoscopic cholecystectomy [26], decontamination skills [27], physical examinations for patients with traumatic head injuries [32], correct positioning for x-ray imaging [33], audiometry techniques [35], nasogastric tube feeding [36], immediate assessment and treatment of patients who are critically ill [37,38], dental anesthesia skills [39], external ventricular drainage [40], and neonatal infection control [41]. Only in 12% (2/16) of the studies was IVR used to teach students anatomical knowledge [30,43], whereas in 6% (1/16) of the studies, IVR was used to orient the students to the setting of the surgical operating room [29].

Most studies (12/16, 75%) featured only a single IVR experience for the students. The exceptions were the study by Bakhos et al [35], which had 3 different IVR cases, and the study by Collaço et al [39], who provided 2 IVR training episodes. Berg and Steinsbekk [37,38] allowed students to self-practice the skills but did not mention the number of sessions. The duration for students to experience IVR learning was typically short, ranging from 5 to 30 minutes in 56% (9/16) of the studies. In total, 38% (6/16) of the studies [25,27,33,37,38,41] allowed students to be exposed to IVR environments as long as they needed to complete the specific tasks or procedures. A total of 6% (1/16) of the studies [39] did not state the exact duration of the students’ IVR learning session.

The IVR products used in the included studies were Oculus VR, Samsung Gear VR, Clinical Education Training Solution VR Clinic, HTC VIVE, and a university-created platform.

Side effects were reported in 25% (4/16) of the studies. Nearly half of the students in the study by Kurul et al [30] reported different forms of slight discomfort (ie, vision discomfort, eyestrain, and general discomfort). In the study by Chao et al [36], 23% of the students reported feeling slightly dizzy, but this did not affect their viewing activities. In contrast, most students in another 12% (2/16) of the studies [26,39] did not experience any side effects.

### Theoretical Frameworks

Learning theory provides a framework to guide the development of teaching activities to help students imbibe, process, and retain the knowledge and skills that they have learned [44,45]. When applied to educational IVR, a learning theory should provide a pedagogical framework and foundation for designing IVR-related teaching and learning strategies. However, most of the included studies (15/16, 94%) did not mention any theoretical approaches underpinning the development of IVR teaching. The exception was the study by Smith et al [27], who used the National League for Nurses Jeffries Simulation Theory [46] as their theoretical basis to guide the design of their teaching innovation. Most studies (15/16, 94%) supplemented the IVR lessons and tutorials by providing additional pedagogical practices or materials to encourage learning. In total, 25% (4/16) of the studies [27,32,34,37] included web-based modules and reading materials in addition to the IVR experience. The provision of either introduction or prebriefing or debriefing sessions before and after IVR learning was mentioned in 44% (7/16) of the studies [25,28,33,35,38,40,41]. A total of 31% (5/16) of the studies [26,29,30,36,39] used IVR as the sole method of learning.

### Risk-of-Bias Assessments

**Figure 2:**
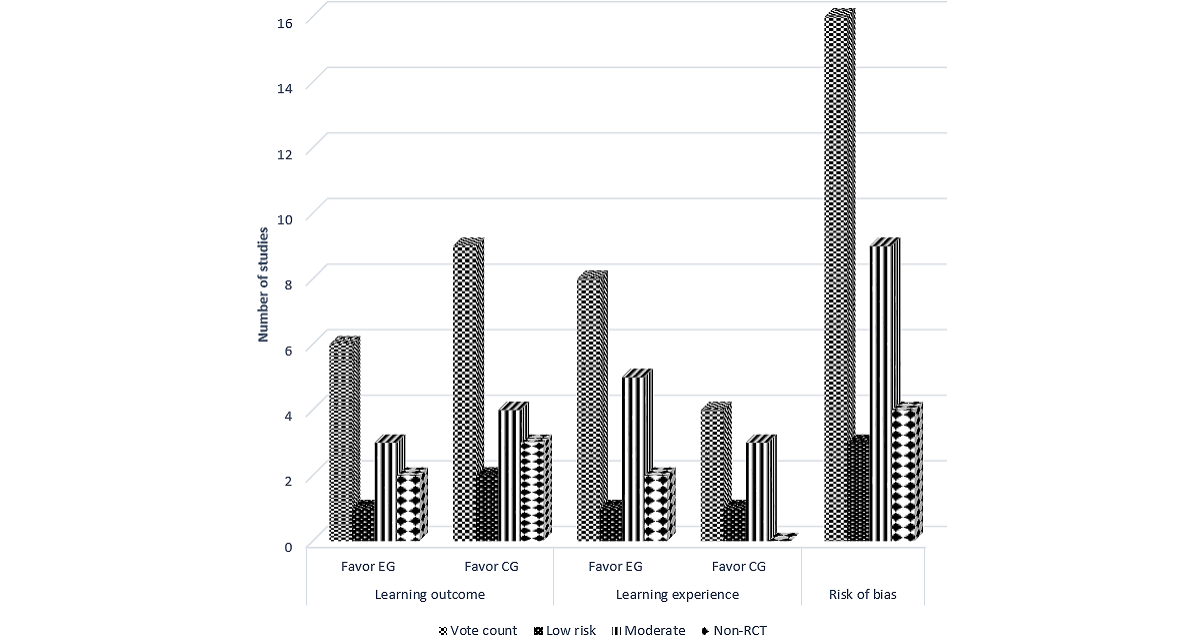
Evidence of effect: direction of effect plot. CG: control group; EG: experimental group; RCT: randomized controlled trial.

Among the included RCTs, a risk of bias was identified in most domains, with the exception of the domains Q7, Q8, and Q10 to Q13 (Table 2). Only 25% (3/12) of the studies [26,28,37,38] gave clear details on the randomization procedure, whereas other studies simply briefly stated that the design was randomized without providing further information. Allocation concealment was not implemented or was unclear in 75% (9/12) of the studies [25,26,28-30,32,34,36,40]. In total, 33% (4/12) of the studies [25,26,33,40] did not report the baseline comparison between the groups, and in 8% (1/12) of the studies [38], there were differences in age and practical experience between the groups. The blinding of participants, intervention providers, and outcome assessors was another major concern with the RCTs. In none of the studies were the participants blinded, and in only 17% (2/12) of the studies [37,38] were both the assessors and intervention providers blinded. Among all 16 evaluated items in the JBI checklist, there were 25% (3/12) of the studies in which 1 to 3 items were viewed as having a low risk of bias and 75% (9/12) of the studies in which 4 to 6 items were viewed as having a moderate risk of bias (Figure 2).

For the quasi-experimental studies, the overall risk of bias was low (Table 3). However, in 25% (1/4) of the studies [27], 13% of the participants failed to complete all the tests, and no detailed explanations were given about which groups were involved and how this issue was handled in the study. In addition, in 50% (2/4) of the studies [35,39], the baseline difference between the groups was not reported.

**Table 2:** Critical appraisal of the included randomized controlled trials (RCTs; Joanna Briggs Institute critical appraisal checklist for RCTs).

Study, year	Q1^a^	Q2^b^	Q3^c^	Q4^d^	Q5^e^	Q6^f^	Q7^g^	Q8^h^	Q9^i^	Q10^j^	Q11^k^	Q12^l^	Q13^m^
Gutiérrez-Maldonado et al [25], 2015	U^n^	N^o^	U	N	U	U	Y^p^	Y	Y	Y	Y	Y	Y
Harrington et al [26,42], 2018	Y	U	U	N	U	U	Y	Y	Y	Y	Y	Y	Y
Zackoff et al [28], 2020	U	U	Y	N	N	U	Y	Y	Y	Y	Y	Y	Y
Francis et al [29], 2020	U	U	Y	N	U	U	Y	Y	Y	Y	Y	Y	Y
Kurul et al [30], 2020	U	U	Y	N	—^q^	Y	Y	Y	Y	Y	Y	Y	Y
Gutiérrez et al [32], 2007	U	N	Y	N	—	U	Y	Y	Y	Y	Y	Y	Y
Pierce et al [31], 2008	U	N	Y	N	—	U	Y	Y	Y	Y	Y	Y	Y
Sapkaroski et al [33], 2019	U	Y	U	N	U	Y	Y	Y	Y	Y	Y	Y	Y
Stepan et al [34], 2017	U	U	Y	N	—	U	Y	Y	Y	Y	Y	Y	Y
Chao et al [36], 2021	U	N	Y	N	N	U	Y	Y	Y	Y	Y	Y	Y
Berg and Steinsbekk [37], 2020	Y	Y	Y	N	Y	Y	Y	Y	Y	Y	Y	Y	Y
Berg and Steinsbekk [38], 2021	Y	Y	N	N	Y	Y	Y	Y	Y	Y	Y	Y	Y
Ros et al [40], 2020	U	U	U	N	U	U	Y	Y	Y	Y	Y	Y	Y

^a^Q1: was true randomization used for assigning participants to treatment groups?

^b^Q2: was the allocation to the treatment groups concealed?

^c^Q3: were the treatment groups similar at baseline?

^d^Q4: were participants blinded to the treatment assignment?

^e^Q5: were those delivering treatment blinded to the treatment assignment?

^f^Q6: were outcome assessors blinded to the treatment assignment?

^g^Q7: were the treatment groups treated identically other than in the intervention of interest?

^h^Q8: was follow-up complete, and if not, were strategies used to address incomplete follow-ups (ie, was there an analysis of patterns of those lost to follow-up)?

^i^Q9: were participants analyzed in the groups to which they were randomized?

^j^Q10: were the outcomes measured in the same way for the treatment groups?

^k^Q11: were the outcomes measured in a reliable way?

^l^Q12: was an appropriate statistical analysis used?

^m^Q13: was the trial design appropriate, and were any deviations from the standard RCT design (individual randomization and parallel groups) accounted for in the conduct and analysis of the trial?

^n^U: unclear.

^o^N: no.

^p^Y: yes.

^q^Not available.

**Table 3:** Critical appraisal of the included quasi-experimental trials (Joanna Briggs Institute critical appraisal checklist for quasi-experimental trials).

Study, year	Q1^a^	Q2^b^	Q3^c^	Q4^d^	Q5^e^	Q6^f^	Q7^g^	Q8^h^	Q9^i^
Smith et al [27], 2018	Y^j^	Y	Y	Y	Y	U^k^	Y	Y	Y
Bakhos et al [35], 2020	Y	U	Y	Y	Y	Y	Y	Y	Y
Collaço et al [39], 2021	Y	U	Y	Y	Y	Y	Y	Y	Y
Yu et al [41], 2021	Y	Y	Y	Y	Y	Y	Y	Y	Y

^a^Q1: are the *cause* and the *effect* made clear in the study (ie, there was no confusion about which variable came first)?

^b^Q2: were the participants who were included in any comparisons similar to each other in any way?

^c^Q3: were the participants who were included in any comparisons receiving similar treatment or care other than the exposure or intervention of interest?

^d^Q4: was there a control group?

^e^Q5: were there multiple measurements of the outcome both before and after the intervention or exposure?

^f^Q6: was the follow-up complete, and if not, were the differences between the groups in terms of their follow-up adequately described and analyzed?

^g^Q7: were the outcomes of the participants included in any comparisons measured in the same way?

^h^Q8: were the outcomes measured in a reliable way?

^i^Q9: was an appropriate statistical analysis used?

^j^Y: yes.

^k^U: unclear.

### Effects of IVR Teaching on Student Learning Outcomes

Of the 16 included studies (reported in 17 papers), 1 (6%) study using IVR for orientation did not include any assessment of student learning outcomes [29]. Therefore, 94% (15/16) of the studies were retained to assess the primary objective (ie, the students’ learning outcomes) based on vote counting and a binomial probability test. Assuming that the true probability of favoring either IVR teaching or non-IVR teaching was equivalent to 0.05 under the null hypothesis (IVR teaching=non-IVR teaching on student learning outcomes), the results showed that 40% (6/15) of the studies [28,30,32,33,39,40] favored IVR teaching (95% CI 16.3%-67.7%; *P*=.61). The remaining 60% (9/15) of the studies [25-27,34-38,41] showed similar effects between IVR and other teaching methods. These figures are below the expected binomial probability mean of 1.60 (SD 0.51) votes. Thus, we need to accept the null hypothesis.

### Procedural Skill Outcomes

In total, 87% (13/15) of the studies adopted IVR to teach procedural skills. A total of 62% (8/13) of the studies [25,27,35-38,41,42] showed that the improvement in the students’ acquisition of procedural skills was similar whether they were in the IVR groups or in the groups that used other teaching methods. In other words, 38% (5/13) of the studies [28,32,33,39,40] indicated that students who received IVR teaching performed significantly better (95% CI 13.9%-68.4%; *P*=.58) than those who received other forms of teaching. These figures are also below the binomial probability mean of 1.62 (SD 0.51) votes; thus, the null hypothesis is accepted.

### Theoretical Knowledge and Other Outcomes

A total of 13% (2/15) of the studies evaluated the effects of IVR on teaching theoretical knowledge [30,34]. IVR teaching was found to have a greater effect on the acquisition of theoretical knowledge compared with the passive control in 50% (1/2) of the studies [30], but in another study [34], the effects of IVR were shown to be similar to those of the passive control. Owing to the small number of studies (only 2 studies), we did not run the binomial test. One study [29] used IVR for orienting students to the operating room, but the students’ knowledge was not tested afterward. In none of the studies was IVR used to enhance the students’ professional attitudes.

### Effects of IVR Teaching on the Students’ Learning Experience

Of the 15 studies, 2 (13%) did not measure students’ learning experience [33,40], 1 (7%) explored students’ satisfaction and experiences with IVR through a focus group (only qualitative data were collected) [27], and 1 (7%) only measured the students’ learning experience in the IVR group but not in the control group [30]. These studies were excluded from the vote counting and binomial test. Of the 12 studies, 8 (67%) favored IVR teaching (95% CI 34.9%-90%; *P*=.59), whereas the remaining 4 (33%) showed similar effects on the students’ learning experience between IVR and other methods of teaching.

In total, 80% (12/15) of the studies [25,26,28,29,32,34-39,41] evaluated the learning experiences of students from both the IVR and control teaching groups. Overall, the students said that they had a more positive experience learning with IVR than with other teaching methods (Table 4). A total of 67% (8/12) of the studies [26,28,29,34-37,41] showed that the students favored IVR teaching, and 33% (4/12) of the studies [25,32,38,39] reported that the learning experiences of students who learned with IVR were similar to those of either the active or passive controls (95% CI 34.9%-90.1%; *P*=.39). These figures are also below the binomial probability mean of 1.33 (SD 0.49) votes; thus, the null hypothesis is accepted. In 17% (2/12) of the studies [27,30], only the experimental group was evaluated, and both studies showed that students liked the approach of using IVR to learn. The effect direction plot of the different studies, together with the associated risk of bias, is shown in Figure 2.

**Table 4:** Effects of immersive virtual reality (IVR) in the included studies (N=16).

Study, year, and country or region	Type of IVR	Outcome measurements	Major findings	Learning outcomes favor group	Learning experience favor group
Gutiérrez-Maldonado et al [25], 2015, Spain	Oculus Rift DK1	Primary outcome: a diagnostic interview skill test; secondary outcome (usability): SUMI^a^	Primary and secondary outcome: the mean score of the EG^b^ was higher than that of the CG^c^, but the difference between the 2 groups was nonsignificant (*P*=.23; *P*=.89).	Similar	Similar
Harrington et al [26,42], 2018, Ireland	Samsung Gear VR^d^	Primary outcome: knowledge retention (8-point multiple choice); secondary outcomes: (1) attentiveness (engagement) and (2) appraisals	Knowledge retention: no significant variances (*P*=.14). Attentiveness (engagement): a crossover analysis revealed a significantly higher level in the EG (*P*<.001) and across periods (*P*<.001) with no significant carryover effect (*P*=.97). Appraisals: two-thirds of participants reported choosing EG.	Similar	EG
Smith et al [27], 2018, United States	Oculus Rift Developer Kit 2	Primary outcomes: (1) decontamination knowledge gain, (2) skill performance, and (3) time spent by students completing the procedure; secondary outcome: qualitative focus group	Primary outcome: decontamination knowledge gain—no significant difference among the 3 groups at all 3 time points; skill performance—no significant difference among the 3 groups at the 2 time points; performance time—no significant difference among the 3 groups at all 3 time points; secondary outcome: high levels of satisfaction in the EG	Similar	Only measured the EG
Zackoff et al [28], 2020, United States	Oculus Rift	Primary outcomes: recognition or interpretation of key examination findings, assignment of an appropriate respiratory status assessment, and recognition of the need to escalate care for patients with impending respiratory failure by using a video-based assessment; secondary outcome: self-assessed competence	Primary outcome: significantly higher in the EG than in the CG; secondary outcome: 81% of the EG demonstrated an improvement in self-assessed competency.	EG	EG
Francis et al [29], 2020, United States	Oculus VR	Self-efficacy of the participants	Self-efficacy significantly improved in the EG (*P*=.007) but not for the CG (*P*=.30).	N/A^e^	EG
Kurul et al [30], 2020, Turkey	3D Organon Anatomy (Medis Media) and 3D glasses (Oculus Rift; Oculus VR)	Primary outcome: written examination; secondary outcome: participants’ perceptions, including enjoyment and learning efficiency	Primary outcome: both the EG and CG had significantly higher posttest scores, but the difference between the pretest results was found to be significantly higher in favor of the EG (*P*<.001). Secondary outcome: 88.8% of students “enjoyed studying anatomy with IVR”; 83.3% of students felt that “it is easy to understand the location of structures with VR.”	EG	Only measured the EG
Gutiérrez et al [32], 2007, and Pierce et al [31], 2008, United States	A platform created at the University of New Mexico	Primary outcome: written examination; secondary outcomes: the efficiency of the user interface and satisfaction with it	Primary outcome: posttest scores were significantly higher than pretest scores in both the EG and CG (within-group). There was a significant interaction between groups and time for the EG. Secondary outcome: there was no overall significant difference in efficiency and satisfaction between the groups.	EG	Similar
Sapkaroski et al [33], 2019, Australia	CESTOL^f^ VR Clinic	Primary outcome: students’ performance in a skill test involving taking images of hand positions with an x-ray machine	The EG performed, on average, 36% (*P*<.001) better in digit separation, 11% (*P*<.001) better in palm flatness, and 23% (*P*<.05) better in central ray positioning onto the third metacarpal. There was no significant difference (*P*=.17) in positioning between the 2 groups.	EG	N/A
Stepan et al [34], 2017, United States	Oculus Rift	Primary outcome: anatomy knowledge quizzes; secondary outcome: participants’ subjective user experience via interview	Primary outcome: no significant difference was found between the EG and CG in the postintervention quiz (*P*=.87) or the retention quiz (*P*=.47). Secondary outcome: subjective learner experience survey—EG were more engaged (*P*<.01), felt more enjoyment (*P*<.01), and thought it was more useful for learning (*P*<.01); IMMS^g^ survey—the EG scored higher in the total IMMS (*P*<.01) and the subscales for attention (*P*<.01), confidence (*P*<.01), and satisfaction (*P*<.01).	Similar	EG
Bakhos et al [35], 2020, France	Oculus Rift 1	Primary outcome: examination of 20 questions; secondary outcome: posttraining satisfaction and self-confidence	Primary outcome: mean posttraining test scores showed greater improvement in the EG, but the difference between the CG and EG was not significant. Secondary outcome: satisfaction and self-confidence ratings were significantly higher for the EG than for the CG.	Similar	EG
Chao et al [36], 2021, Taiwan	HTC VIVE	Primary outcome: nasogastric tube feeding quiz; secondary outcomes: (1) confidence scale and (2) satisfaction	There were no differences in knowledge (*P*=.84) and confidence (*P*=.96) between the 2 groups. Within groups, the scores on knowledge and confidence improved significantly in both the EG and CG immediately and 1 month after the intervention (*P*<.01). There was a significant difference in satisfaction levels between the intervention and comparison groups (*t*=2.30; *P*=.03).	Similar	EG
Berg and Steinsbekk [37], 2020, Norway	Oculus Rift S or Oculus Quest	Primary outcome: participants’ knowledge and performance of the ABCDE^h^ approach (skill test); secondary outcome: students’ experiences	Primary outcome: noninferiority of the individual IVR conducting all observations in the correct order (EG vs CG: 24.8% vs 27.1%; absolute difference: 2.3% points, one-sided 95% CI 2.3%-10.8%); secondary outcomes were similar between the groups, but more students in the EG reported liking the way they practiced and stated that it was a good way to learn. The EG also scored high on the System Usability Scale.	Similar	EG
Berg and Steinsbekk [38], 2021, Norway	Oculus Rift S or Oculus Quest	Primary outcome: participants’ knowledge and performance of the ABCDE approach (skill test); secondary outcome: participants’ experience	Primary outcome: 29 (20%) participants in the EG and 30 (21%) participants in the CG answered everything correctly. Knowledge and performance of the ABCDE approach were similar in the 2 groups of students, except that the EG performed better in the report on respiratory frequency and in the usability test. Secondary outcome: the EG were more displeased about the learning experience, but the difference was not significant.	Similar	Similar
Collaço et al [39], 2021, Brazil	Samsung	Primary outcome: skill test; secondary outcome: the participants’ perceptions	Primary outcome: for the execution time—NP^i^ and NT^j^ took significantly longer than the full (EG) and NH^k^ (*P*<.001) groups. The full (EG) and NH groups were more accurate in needle insertion than the NP and NT (*P*<.001). There were no significant differences among the groups in needle angle (*P*=.44) or needle depth (*P*=.24). Secondary outcome: no significant differences were found among the groups for factor 1 and factor 3. For factor 2, the NT group reported significantly (*P*<.001) more difficulty than the other groups (factor 1: tactile realism; factor 2: syringe control; factor 3: ease of performance).	EG	Similar
Ros et al [40], 2020, France	Samsung Gear VR	Primary outcome: written test about indications, patient management, and preparation until the incision	The EG had significantly better results (*P*=.01) in answering the questionnaire compared with the CG. The results were similar at 6 months (the scores in the EG were higher than in the CG but were nonsignificant).	EG	N/A
Yu et al [41], 2021, Korea	HTC VIVE	Student knowledge (HirNICCS^l^), self-efficacy (10-point scale), and satisfaction (5-point scale)	There was no significant difference between the EG and CG (*P*=.21) in knowledge. The EG showed a greater increase in self-efficacy than the CG(*P*=.02). The EG had a higher satisfaction score than the CG (*P*<.001).	Similar	EG

^a^SUMI: Software Usability Measurement Inventory.

^b^EG: experimental group.

^c^CG: control group.

^d^VR: virtual reality.

^e^N/A: not applicable.

^f^CESTOL: Clinical Education Training Solution.

^g^IMMS: Instructional Materials Motivation Survey.

^h^ABCDE: airways, breathing, circulation, disability, exposure.

^i^NP: nonpreceptorship.

^j^NT: nontraining.

^k^NH: nonhaptic feedback.

^l^HirNICCS: High-Risk Neonatal Infection Control Competency Scale Knowledge.

### Certainty of Evidence

The generated GRADE evidence profile was used to present a synthesis of the findings regarding objective 1 (ie, students’ learning outcomes) and objective 2 (ie, students’ learning experiences) in Table 5. As there were serious concerns about most of the studies with regard to the study design, inconsistent results, and a strong suspected publication bias, all the evidence was considered to have a very low level of certainty.

**Table 5:** Grading of Recommendations Assessment, Development, and Evaluation evidence profile: certainty of evidence for the learning outcomes and learning experiences (N=16).

Outcome	Studies, n (%)	Study design	Risk of bias	Inconsistency	Indirectness	Other considerations	Summary of findings				Certainty
							EG^a^	CG^b^	Direction		
Students’ learning outcomes (assessed via examination and skill tests)	15 (94)	Randomized trials	Serious^c^	Serious^d^	Not serious	Publication bias strongly suspected^e^	6	9	EG<CG	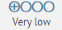	
Students’ learning on procedural skill outcomes (assessed via examination and skill tests)	13 (81)	Randomized trials	Serious^f^	Serious^g^	Not serious	Publication bias strongly suspected^e^	5	8	EG<CG	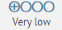	
Students’ learning on theoretical knowledge and other outcomes (assessed via examination and skill tests)	2 (12)	Randomized trials	Serious^h^	Serious^i^	Not serious	Publication bias strongly suspected^e^	1	1	EG=CG	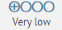	
Students’ learning experience (assessed via questionnaires)	12 (75)	Randomized trials	Serious^j^	Serious^k^	Not serious	Publication bias strongly suspected^e^	8	4	EG>CG	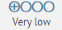	

^a^EG: experimental group.

^b^CG: control group.

^c^A total of 27% (4/15) of the studies that evaluated the students’ learning outcomes were quasi-experimental trials. According to the Joanna Brigs Institute critical appraisal checklist for randomized controlled trials, the remaining 11 randomized controlled trials had 1 to 6 items that were rated at *no* or *unclear*, which indicates that there were issues in the study design leading to a serious risk of bias.

^d^A total of 40% (6/15) of the studies favored immersive virtual reality teaching (*P*=.10 in a binomial probability test showing that the null hypothesis is accepted).

^e^None of the papers registered their study protocol; therefore, we could not check if there was any publication bias.

^f^A total of 31% (4/13) of the studies were quasi-experimental trials. According to the Joanna Brigs Institute critical appraisal checklist for randomized controlled trials, the remaining 9 randomized controlled trials had 1 to 6 items that were rated at *no* or unclear, which indicates that there were issues in the study design leading to a serious risk of bias.

^g^Only 38% (5/13) of the studies favored immersive virtual reality teaching (*P*=.58 in a binomial probability test showing that the null hypothesis is accepted).

^h^According to the Joanna Brigs Institute critical appraisal checklist for randomized controlled trials, these 3 randomized controlled trials have 3 to 5 items rated at *no*” or *unclear*, which indicates issues in the study design.

^i^A total of 50% (1/2) of the studies favored immersive virtual reality teaching. One study used IVR to orient the students to the operating room.

^j^A total of 17% (2/12) of the studies were quasi-experimental trials. According to the Joanna Brigs Institute critical appraisal checklist for randomized controlled trials, the remaining 10 randomized controlled trials had 1 to 5 items rated at *no* or *unclear*, which indicates issues in the study design leading to a serious risk of bias.

^k^A total of 62% (8/13) of the studies favored immersive virtual reality teaching (*P*=.39 in a binomial probability test showing that the null hypothesis is accepted).

## Discussion

### Principal Findings

The findings of this systematic review demonstrate that the use of IVR teaching in undergraduate health care education is effective in enhancing the procedural skills and knowledge acquisition of students. However, the effects on these learning outcomes were similar to those of other teaching approaches such as desktop-based VR or conventional classroom teaching. A vote count showed that 60% (9/15; 95% CI 16.3%-67.7%; *P*=.61) of the studies identified similar learning outcomes between IVR teaching and other teaching approaches regardless of the teaching domain.

In general, in the 15 studies, the students indicated that they had positive learning experiences with IVR teaching, including increased satisfaction, self-confidence, self-assessed competency, self-efficacy, and enjoyment with IVR teaching. When compared with those in the control group who received other methods of teaching, the vote count showed that, in 62% (8/13) of the studies, the participants were in favor of using IVR as a teaching medium. However, the results of the binomial test (95% CI 34.9%-90%; *P*=.59) did not indicate a statistically significant difference. Therefore, the results indicated similarly positive learning experiences between students who received IVR and those who received other teaching approaches. Considering the low-level evidence identified based on the GRADE tool, it is inconclusive whether IVR teaching is superior to other forms of VR and to conventional teaching methods with regard to students’ learning outcomes and learning experiences.

### Comparison With Prior Work

Although the application of IVR in health care teaching has become more prevalent in recent years, only a limited number of systematic reviews have focused solely on evaluating its effects. Many reviews have included all forms of VR teaching (such as 2D computer games). Therefore, the effects of IVR on teaching are yet to be confirmed. This review is one of the few to provide additional evidence on the effects of IVR in health care teaching.

The inconclusive findings on the students’ learning outcomes identified in this review are similar to those of the systematic review of 29 articles by Hamilton et al [47]. Their review also reported that the effects of IVR teaching on learning outcomes and attainment levels were inconsistent compared with those of conventional desktop-based VR or the original physical training scenario [47]. These findings contradict those of the systematic review of 17 articles by Mao et al [11]. Their review reported that medical students who received IVR training performed significantly faster in the time required to complete surgical procedures and had higher scores on procedural checklists than those who received other forms of training [11]. A possible reason for the inconsistent findings may be the different target populations and objectives of the studies. Both our review and the review by Hamilton et al [47] included studies targeting the teaching of theoretical knowledge and training in procedural skills, but the review by Mao et al [11] focused mainly on training in surgical procedural skills. It is possible that IVR may be more effective for teaching procedural skills as it provides real-world simulations to give the students an immersive training experience and allows students to repeatedly practice the same procedures. However, when used in theoretical teaching, the instillation of knowledge appears to rely more on personal memorization and understanding [48].

The lack of teaching theories and pedagogies to guide the integration of IVR into health care education may be another reason for the inconsistent teaching outcomes shown in this review. This finding is also similar to that of the review by Hamilton et al [47], which identified only 1 study with a pedagogical framework to guide the development of IVR teaching [47]. Among all the included studies in this review, only the study by Smith et al [27] adopted the National League for Nurses Jeffries Simulation Theory to guide the design of simulation teaching, which is that students learn information as part of a simulated experience [46]. In general, the included studies indicated that their students’ exposure to IVR learning experiences was short. A total of 75% (12/16) of the studies arranged a single IVR learning experience ranging from 5 to 30 minutes for their students. To maximize the impacts of IVR teaching, it has been suggested that educators incorporate IVR teaching into their courses guided by a pedagogy to enrich the teaching contexts with actual experience, insightful reflections, realistic practices, and real-world connections [49].

Although the learning outcomes from IVR teaching are comparable with those from other teaching approaches, IVR still has many advantages in undergraduate health care education. In particular, it can improve the students’ positive learning experiences. In total, 62% (10/16) of the studies in this review found that students favored learning with IVR, which can motivate them to learn actively rather than passively, although the results of the binomial test did not indicate a statistically significant difference. Similarly, the systematic review by Mao et al [11] reported that IVR could improve the self-confidence of medical trainees in performing surgical procedures compared with other training methods. In addition, IVR teaching can incorporate different scenarios of patients in critically ill and emergency situations, giving students valuable hands-on opportunities in a safe and controlled environment. In this review, 31% (5/16) of the studies [36-39,41] simulated real-world scenarios to train students in clinical skills such as tube feeding and conducting physical assessments of infants with respiratory distress. This provided the students with a safe environment in which to avoid unnecessary adverse events, which health care students often experience.

Although there is evidence showing that IVR can be used to enhance the professional attitudes of students, such as in the areas of empathy, decision-making, or collaborative teamwork, none of the studies in this review focused on this domain of teaching. Most (13/16, 81%) used IVR to train students in procedural skills, 12% (2/16) used it to teach anatomy, and 6% (1/16) used it for orientation. A review of 178 medical studies showed that using IVR in teaching could effectively improve medical students’ understanding of the impacts of gerontological diseases on the daily life of older adults. Moreover, after learning through IVR, the students expressed more feelings of empathy toward older adults with sensory impairments and dementia [50]. Another study showed that IVR teaching can create scenarios that closely resemble those in real health care settings, allowing students to immerse themselves in practicing clinical reasoning and learn how to deal with emergencies, which can boost their ability to make decisions and determine priorities [43]. Another study tested the awareness and decision-making abilities of novice surgical residents by using IVR in surgical training. The results showed that the residents’ scores improved significantly in comparison with the scores of those who received conventional PowerPoint teaching [51]. In addition, IVR teaching can help students build their communication skills. A study found that IVR teaching could significantly promote the communication skills of pediatricians to persuade parents of the merits of the influenza vaccine injection [52]. Good communication skills are a core attribute of clinical competence, and IVR teaching can allow students to practice this skill in VR clinical settings and enable them to learn how to deal with different situations [53].

This review has implications for both research and educational practice. First, the unique capabilities of IVR teaching are yet to be fully exploited. In addition to training in procedural skills and inputting theoretical knowledge, IVR teaching has great potential to be used for other teaching domains such as health care professional attitudes. Second, health educators need to align pedagogy with IVR teaching for successful integration into undergraduate health care education [54]. Using pedagogy to guide the integration of IVR teaching into a course can maximize the impact on the students’ learning outcomes by enriching teaching contexts with robust instructions, realistic practices, and real-world connections [49]. Third, it is extremely important in undergraduate health care training for skills and knowledge to be retained and applied in real clinical settings. Therefore, in addition to immediate skill tests or written examinations, other forms of assessment such as essays or group discussions can be considered for an in-depth evaluation of the students’ understanding of the course [47]. It may be difficult to measure the differences between IVR and traditional teaching in terms of the transferability from knowledge to actual clinical practice. It is hard to tell whether students have transferred what they have learned from IVR into their clinical practice. Current evaluation methods focus on superficial and short-term effects. Clinically based observational studies conducted over a long period can be considered in the future. Finally, the quality of the current studies, especially the RCTs, is a concern. Standardized guidelines such as CONSORT (Consolidated Standards of Reporting Trials) [55] should be referred to when designing a study.

### Strengths and Limitations

Our review provides the most up-to-date evidence on the positive effects of IVR in undergraduate health professional education. We conducted a comprehensive search across different databases and followed the Cochrane gold-standard methodology together with a meta-synthesis while conducting this systematic review. This systematic review has some limitations. First, only articles published in English were included. It is possible that articles in other languages were overlooked. However, we adopted relatively broad eligibility criteria for the study types (RCTs and quasi-experimental trials), which led to sufficient findings. Second, the heterogeneity of the included studies prevented the data from being pooled for a meta-analysis, meaning that the effect size of the IVR on teaching outcomes could not be determined. Third, we did not analyze the learning experiences of students from data collected using qualitative methods. Although a qualitative method was only mentioned in 6% (1/16) of the included studies, it is possible that some findings related to the subjective learning experiences of students with IVR were missed. Fourth, we originally hypothesized that the types of equipment with different levels of immersive experiences used for IVR teaching would affect students’ learning outcomes and experiences. However, we found no studies that measured the level of immersive experience according to the type of VR equipment. Therefore, we could not perform any subgroup analyses to investigate these possible moderating effects on students’ learning outcomes and experiences. However, in our review, we found no evidence to support this hypothesis. The use of equipment played a small role in influencing teaching and learning outcomes. Finally, this review only focused on undergraduate health care students; therefore, the findings may not be transferable to students in other majors.

### Future Directions

Despite the positive effects of IVR when adopted in undergraduate health care education in the digital era, robust assessments through high-quality, large-scale studies with long follow-up periods are still lacking. In addition, the true efficacy of IVR is best assessed through long-term integration into a real-world training guide with a pedagogical framework to maximize the effects of IVR on education. In addition to procedural training and theoretical knowledge, IVR has the potential to be used to train students in other desirable attributes of health care professionals (such as communication skills, decision-making skills, and feelings of empathy toward patients). Given its positive impacts on students’ learning outcomes and experiences, we recommend further investigation with rigorous studies focusing on important outcomes for students following long-term incorporation into different health care curricula in different teaching domains.

### Conclusions

This systematic review demonstrates that the use of IVR teaching in undergraduate health care education is effective in enhancing the procedural skills and knowledge acquisition of students, although the effects on these learning outcomes were similar to those of other teaching approaches. IVR also has an advantage in enhancing the positive learning experiences of students. In total, 50% (8/16) of the studies in this review indicated that the students favored IVR teaching over other teaching methods. IVR teaching also has great potential to be used in other teaching domains such as enhancing the professional attitudes of students. Unfortunately, none of the included studies focused on those teaching domains, which are worth exploring in future studies. Finally, health educators should align pedagogy with IVR teaching for successful integration into undergraduate health care education to maximize the impact on students’ learning outcomes.

## Appendix

Multimedia Appendix 1 PRISMA (Preferred Reporting Items for Systematic Reviews and Meta-Analyses) checklist.

Multimedia Appendix 2 Search strategy for different databases.

## Abbreviations

CONSORT: Consolidated Standards of Reporting Trials
GRADE: Grading of Recommendations Assessment, Development, and Evaluation
IVR: immersive virtual reality
JBI: Joanna Briggs Institute
PICOS: Population, Intervention, Comparison, Outcome, and Study Design
PRISMA: Preferred Reporting Items for Systematic Reviews and Meta-Analyses
RCT: randomized controlled trial
SWiM: Synthesis Without Meta-analysis
