# Supplementary material for: The Effects of Immersive Virtual Reality Applications on Enhancing the Learning Outcomes of Undergraduate Health Care Students: Systematic Review With Meta-synthesis
Source: J Med Internet Res. 2023 Mar 6;25:e39989. doi: 10.2196/39989 (PMC10028520; doi:10.2196/39989)
Supplement: Multimedia Appendix 2 [file jmir_v25i1e39989_app2.docx]

**Multimedia Appendix 2. Search strategy for different databases.**

**a. Search terms**

| Key Concepts | Search Terms |
| --- | --- |
| Healthcare undergraduate | ((Healthcare OR Health OR Health Personnel OR Allied Health) AND (Students OR Undergraduates OR Trainee)) OR (Medical Students OR Nursing Students OR Dietetics Students OR Radiologic Technology Students OR Physical Therapy Students OR Occupational Therapy Students) |
| Immersive Virtual Reality | Immersive Virtual Reality OR (Virtual Reality AND (immers* OR submers*)) OR (Virtual World AND (immers* OR submers*)) OR (Simulated Environment AND (immers* OR submers*))  OR (Virtual Environment AND (immers* OR submers*)) OR (Simulation AND (immers* OR submers*)) |
| Learning Outcome | Knowledge OR Skill OR Psychomotor Skill OR Non-psychomotor Skill OR Skill Training OR Technical Skill OR Non-technical Skill OR Affective Reaction OR Attitude OR Learning Experience OR Learning Outcome OR Satisfactory OR Perception OR Communication skill |

**b: Searching strategy in databases:**

**PubMed**

#1 "Virtual Reality"[Mesh] AND immersive

#2 Immersive Virtual Reality OR (Virtual Reality AND (immers* OR submers*)) OR (Virtual World AND (immers* OR submers*)) OR (Simulated Environment AND (immers* OR submers*)) OR (Virtual Environment AND (immers* OR submers*)) OR (Simulation AND (immers* OR submers*))

#3 #1 OR #2

#4 "students, health occupations"[MeSH Terms])

#5 (Healthcare OR Health OR Health Occupations OR Allied Health) AND (Students OR Undergraduates OR Trainee)

#6 #4 OR #5

#7 #3 AND #6

**Medline**

#1 ((MH "Virtual Reality") AND (MH "Immersion")) OR "immersive virtual reality"

#2 TX Immersive Virtual Reality OR (Virtual Reality AND (immers* OR submers*)) OR (Virtual World AND (immers* OR submers*)) OR (Simulated Environment AND (immers* OR submers*))  OR (Virtual Environment AND (immers* OR submers*)) OR (Simulation AND (immers* OR submers*))

#3 #1 OR #2

#4 (MH "Students, Premedical") OR (MH "Students, Pharmacy") OR (MH "Students, Nursing") OR (MH "Students, Medical") OR (MH "Students, Dental") OR (MH "Students, Health Occupations") OR (MH "Students, Public Health")

#5 TX (Healthcare OR Health OR Health Occupations OR Allied Health) AND (Students OR Undergraduates OR Trainee)

#6 #4 OR #5

#7 #3 AND #6

**EMBASE**:

#1 'health student'/exp

#2 'immersive virtual reality'/exp

#3 'computer simulation'/exp AND (immers* OR submers*)

#4 #2 OR #3

#5 ((healthcare OR health) AND service AND occupations OR allied) AND health AND (students OR undergraduates OR trainee)

#6 ((((immersive AND reality or  AND virtual AND reality AND (immers* OR submers*) OR virtual) AND world AND (immers* OR submers*) OR simulated) AND environment AND (immers* OR submers*) OR virtual) AND environment AND (immers* OR submers*) OR simulation) AND (immers* OR submers*)

#7 #1 OR #5

#8 #4 OR #6

#9 #7 AND #8

**Scopus**

#1 ( "immersive virtual reality" OR ( "virtual reality" AND ( immers* OR submers* ) ) OR ( "virtual world" AND ( immers* OR submers* ) ) OR ( "simulated environment" AND ( immers* OR submers* ) ) OR ( "virtual environment" AND ( immers* OR submers* ) ) OR ( simulation AND ( immers* OR submers* ) ) )

#2 ( ( healthcare OR "Health Occupations" OR "Allied Health" ) AND ( students OR undergraduates OR trainee ) )

#3 ( LIMIT-TO ( SUBJAREA , "MEDI" ) OR LIMIT-TO ( SUBJAREA , "NURS" ) )

#4 ( LIMIT-TO ( LANGUAGE , "English" ) )

#5 #1 AND #2 AND #3 AND #4
